# Analysis and Functional Annotation of Expressed Sequence Tags from the Asian Longhorned Beetle, *Anoplophora glabripennis*

**DOI:** 10.1673/031.009.2101

**Published:** 2009-05-20

**Authors:** Wayne B. Hunter, Michael T. Smith, Laura E. Hunnicutt

**Affiliations:** ^1^ U.S. Department of Agriculture, Agricultural Research Service, U.S. Horticultural Research Laboratory, Ft. Pierce, Florida 34945; ^2^ U.S. Department of Agriculture, Agricultural Research Service, Beneficial Insects Introduction Research Unit, Newark, Delaware 19713; ^3^ Current address: North Carolina State University, Raleigh, North Carolina 27695

**Keywords:** Coleoptera, development, EST, insect, morphogenesis, transcriptome

## Abstract

The Asian longhorned beetle, *Anoplophora glabripennis* (Motschulsky) (Coleoptera: Cerambycidae), is one of the most economically and ecologically devastating forest insects to invade North America in recent years. Despite its substantial impact, limited effort has been expended to define the genetic and molecular make-up of this species. Considering the significant role played by late-stadia larvae in host tree decimation, a small-scale EST sequencing project was done using a cDNA library constructed from 5^th^ -instar *A. glabripennis*. The resultant dataset consisted of 599 high quality ESTs that, upon assembly, yielded 381 potentially unique transcripts. Each of these transcripts was catalogued as to putative molecular function, biological process, and associated cellular component according to the Gene Ontology classification system. Using this annotated dataset, a subset of assembled sequences was identified that are putatively associated with *A. glabnpennis* development and metamorphosis. This work will contribute to understanding of the diverse molecular mechanisms that underlie coleopteran morphogenesis and enable the future development of novel control strategies for management of this insect pest.

## Introduction

The Asian longhorned beetle, *Anoplophora glabripennis* (Motschulsky) (Coleoptera: Cerambycidae), is a pest native to eastern China and Korea (Lingafelter and Hoebeke 2002). In 1996, this insect was introduced into the United States, presumably via wood packing materials used to import cargo from Asia (Smith 2003). Since its initial discovery in the state of New York, infestations have been detected in Illinois (Poland et al. 1998), New Jersey (Haack 2003), and the province of Ontario, Canada (CFIA 2005; Haack 2006). *A. glabripennis* grow and reproduce on an array of hardwoods including members of the genera *Acer* (maple), *Aesculus* (horsechestnut), *Betula* (birch), *Celtis* (hackberry), *Plantanus* (plant tree, sycamore), *Populus* (poplar), *Salix* (willow), and *Ulmus* (elm) (Sawyer 2003; Ric et al. 2006). Late-instar grubs are especially destructive, forging winding galleries into the heartwood of the tree. This feeding behavior causes branch dieback and, in cases of heavy or persistent infestations, can result in structural deterioration and often tree mortality (Haack et al. 1997).

Burgeoning globalized trade presents a serious challenge in that *A. glabripennis* now have the opportunity to infiltrate via multiple points of entry, mitigating the efficacy of the small number of quarantine facilities currently in place. Undetected, this devastating pest could disseminate throughout regions of North America where suitable host trees exist. Nowak et al. (2001) estimated that, if this occurs, up to 1.2 billion urban shade trees with a compensatory value of $669 billion could be lost. While substantial, these figures do not factor in collateral losses such as degraded aesthetics and lowered property values nor do they take into account the potential impact to both commercial and natural forest stands.

At present, eradication efforts center on the identification and removal of trees showing signs of *A. glabripennis* infestation. As of 2002, $110.9 million was expended by federal, state, and city governments in New York and Illinois as part of this program (Stewart 2002). To gauge the utility of systemic insecticides as a supplement to this effort, scientists from the USDA Forest Service performed field evaluations in which trees were treated with either imidacloprid or thiocloprid. While successful in reducing *A. glabripennis* populations, neither compound provided complete control (Poland et al. 2006).

Only recently has research begun to shift focus from chemical-based control strategies to the development of sustainable biocontrol alternatives including entomopathogenic fungi, rhabditoid nematode species, microsporidia, natural predators and parasitoids (Smith et al. 2002, and references therein; Hajek et al. 2006) as well as artificial lures and bait/trap tree systems (Li et al. 1999; Zhang et al. 2002). Furthermore, nominal effort has gone into the investigation of genome-based approaches for management of *A. glabripennis*. To facilitate this work, our laboratory conducted a small-scale EST sequencing project and posted preliminary data to the National Center for Biotechnology Information (NCBI) dbEST where it is freely accessible to the scientific community. Because of the significant role played by late-stadia larvae in host tree decimation, 5^th^ -instar *A. glabripennis* were selected as a base for the transcriptome survey described herein.

## Materials and Methods

### Insects

Fifth-instar *A. glabripennis* were obtained from a colony managed by Michael Smith at the USDA ARS Beneficial Insects Introduction Research Unit (Newark, DE). Insects were maintained as previously described by Dubois et al. (2002). Larvae were ground directly in guanidineisothiocyanate buffer (1 larva per 20 ml buffer) and stored at -40°C prior to shipment.

### RNA extraction and library construction

Upon arrival at the USDA ARS U.S. Horticultural Research Laboratory (Ft. Pierce, FL), the majority of samples were transferred to an ultra-low temperature freezer (-80°C) for archival purposes and a single larva was subjected to further processing. Buffer RLT (Qiagen, www.qiagen.com) was added to the primary sample at 2.5X the original volume along with 150 µl β-mercaptoethanol. The sample was placed at -40°C for 10 min then incubated at 37°C for 20 min. Intact tissues were further homogenized with a QIAShredder® and total RNA extracted using an RNeasy® Maxi Kit (Qiagen) according to the manufacturer's instructions. The eluate was precipitated in 0.1 volumes 3M sodium acetate and 2.5 volumes absolute ethanol at -4O°C overnight and the resultant pellet resuspended in 35 µl RNase-free water. Poly(A)+ RNA was purified using the MicroPoly(A)Pure Kit (Ambion, www.ambion.com). A primary library was constructed with Stratagene's ZAP-cDNA® Library Construction Kit (Stratagene, www.stratagene.com) and subsequently mass excised using ExAssist® Helper Phage (Stratagene). The library had a titer of 9.75 × 105 colony forming units per ml with inserts averaging ∼ 1,221 bp. Transformants were recovered by random colony selection and grown overnight at 32°C, 125 rpm in LB Broth supplemented with 100 mg/ml ampicillin.

### EST sequencing

Plasmid DNA was extracted using the Qiagen Liquid Handling Robot (Model 9600) in conjunction with the QIAprep 96 Turbo Miniprep Kit according to the recommended protocol. Single-pass sequencing was performed using the ABI PRISM® BigDye™ Primer Cycle Sequencing Kit (Applied Biosystems, www.appliedbiosystems.com) and a universal T3 primer. Reaction products were precipitated, resuspended in 15 µl sterile water, and loaded onto an ABI 3730 DNA Analyzer (Applied Biosystems).

### Sequence analysis

Base calling was performed by TraceTuner™ (Paracel, www.paracel.com) and low-quality bases (quality score <20) were stripped from both ends of each EST. Quality trimming, vector trimming, and sequence fragment alignments were executed using Sequencher™ software (Gene Codes, www.genecodes.com). Sequencher contig assembly parameters were set using a minimum overlap of 50 bp and 90% identity. Contigs joined by vector sequence were flagged for possible miss-assembly and manually edited. The EST sequences reported in this study have been deposited in GenBank's dbEST under accession numbers DR108748-DR109303.

### Sequence annotation and Gene Ontology classification

Putative sequence identity was determined based on BLAST similarity searches using the NCBI BLAST server (www.ncbi.nlm.nih.gov) with comparisons made to both non-redundant nucleic acid and protein databases using BLASTN and BLASTX, respectively. Matches with an E-value ≤-10 were considered significant and were classified according to the Gene Ontology classification system. In the case of CG numbers (e.g., CG30437-PA), annotations were conferred using the associated CV term provided by FlyBase (www.flybase.org). All other sequences were associated with a molecular function, biological process, and cellular component based on searches to the Gene Ontology database (www.geneontology.org). Custom Perl scripts and Excel spreadsheets were used for BLAST parsing and table generation. The SignalP 3.0 Server was used to predict the presence and location of signal peptide cleavage sites (www.cbs.dtu.dk/services/SignalP/).

**Table 1.**
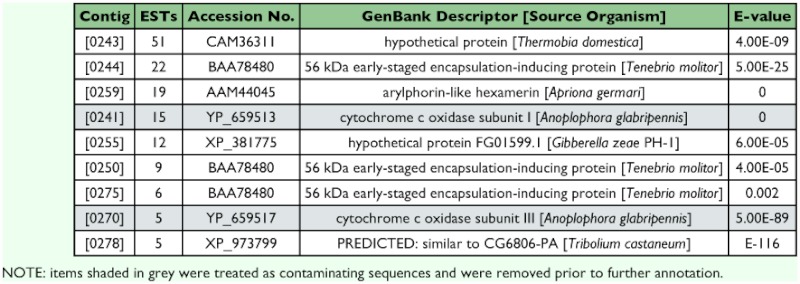
Most abundantly represented transcripts in the *A. glabripennis* cDNA library

## Results and Discussion

### General overview

A single *A. glabripennis* larva was used for this study so that allelic variations within an individual (EST allele counts) could be distinguished from those that may exist across a population (population allele frequency). 5'-end one-pass sequencing of the cDNA library yielded 672 ESTs, of which 599 were designated as high quality (i.e., ≥200 bases with a TraceTuner™ score of 20 or better). ESTs ranged in size from 206 to 828 bases with an average length of 650 bases. Upon assembly, these sequences were condensed to form 47 contiguous sequences (contigs), leaving 334 as singletons. Contigs and singlets together culminated in 381 unique sequences that putatively represent distinct transcripts. Contigs ranged in size from 392 to 2,240 bases with an average length of 954 bases; whereas, singletons varied from 206 to 828 bases with an average length of 647 bases.

### Highly redundant transcripts

The calculated redundancy of the library was ∼32% with nine contigs found to be highly redundant (i.e., containing ≥5 ESTs; Table 1) accounting for 24% of the total ESTs. Two of the contigs, representing 20 ESTs, had significant sequence similarity to mitochondrial genes and were subsequently discarded from the transcriptome survey. Nearly half of the highly redundant contigs had no significant similarity (E >-10) to any sequence listed within NCBI's nr database. These transcripts correspond to potentially novel genes specific to *A. glabripennis* and warrant further examination. The remaining three contiguous sequences returned significant matches to proteins previously identified in other coleopteran species. The most frequently represented of these transcripts, WHALB [0244], constituted 22 ESTs and matched most closely to a 56 kDa early-staged encapsulation-relating protein previously identified from *Tenebrio molitor* larvae. Upon assessment of alignment integrity, two sequence variants differing by 14 single nucleotide polymorphisms (SNPs) were resolved. Consequently, WHALB [0244] was dissolved and realigned to form two assembled sequences, WHALB [0244a] containing 10 ESTs and WHALB [0244b] containing 12 ESTs. Both variants possessed a single open reading frame (ORF) consisting of 456 amino acid residues, the first 15 of which are thought to encode a leader/signal peptide. Amino acid abundance analyses of the translated protein sequences revealed a preponderance of Gln (99 residues or 22%), Gly (46–47 residues or 10%), and Leu (45 residues or 10%) within the coding domain while Cys and His levels were negligible (<0.5%). Although comparable to the cDNA of *T. molitor* 56-kDa encapsulation-relating protein with respect to amino acid abundance and overall sequence similarity, significant distinctions were noted including an eight amino acid insertion shortly after the signal peptide and 11 deletions scattered along the length of the coding domain (Cho et al. 1999). This would seem to indicate that WHALB [0244a] and WHALB [0244b] represent novel proteins which may play a role in *A. glabripennis* cellular defense. As such, both coding domains have been deposited into GenBank under accession numbers EF583868 and EF583869. The second most highly redundant contig, WHALB [0259], contained 19 ESTs and appeared to span the coding region for an arylphorin-like hexameric storage protein, denoted AglHEX (accession number EF583870). AglHEX had an ORF of 2,151 nucleotides, encoding a protein precursor 717 amino acids in length. The N-terminal of this precursor most likely contains a cleavage site between AYS_17_/A_18_V, indicating a signal peptide for transmembrane transport. In addition, the following highly conserved larval storage protein (LSP) signature sequence patterns were noted: LSP signature-1 motif Y(F/Y/W)XED(L/I/V/M)X_2_NX_6_HX_3_P) and LSP signature-2 motif TX_2_RDPX(F/Y)(F/Y/W) with the corresponding sequences in AglHEX as YYLEDVGLNAFYYYYHLYYP_218–237_ and TSMRDPVF_421–428_ (Zhu et al. 2002). Contig WHALB [0278], comprised of five ESTs, showed greatest sequence similarity to a predicted protein from *Tribolium castaneum* annotated as similar to *Drosophila melanogaster* CG6806-PA. When queried to FlyBase, it was determined that this transcript also corresponded to a LSP [partial LSP-2; ∼400 amino acids missing from the protein's N-terminal]. Of 307 in-frame residues, WHALB [0278] contained five Met (2%) and 48 aromatic amino acids (16%), a composition indicative of arylphorin-like storage proteins (Telfer and Kungel 1991).

### Functional classification of 5^th^-instar *A. glabripennis* ESTs

A BLASTN search of the entire dataset revealed six contigs and five singletons with significant sequence similarity either to non-nuclear transcripts (e.g., rRNA genes and mitochondrial genes) or contaminating organismal transcripts (e.g., transcripts of plant, bacterial, or trematode origin). These assembled sequences, representing 34 ESTs, were removed from the dataset prior to further analysis.

A total of 258 sequences (29 contigs and 229 singletons; 58% ESTs) showed significant sequence similarity to known proteins. Four sequences (3 contigs and 1 singleton; 4% ESTs) had hits with E-values ≥10^-150^, 35 sequences (9 contigs and 26 singletons; 8% ESTs) had hits with E-values between 10^-100^ and 10^-149^, 114 sequences (11 contigs and 103 singletons; 22% ESTs) had hits with E-values between 10^-50^ and 10^-99^, 48 sequences (3 contigs and 45 singletons; 9% ESTs) had hits with E-values between 10^-30^ and 10^-49^ , and 56 sequences (3 contigs and 53 singletons; 14% ESTs) had hits with E-values between 10^-10^ and 10^-29^ . The remainder of the sequences (9 contigs and 85 singletons; 42% ESTs) failed to return meaningful matches (E >-10). The best match (i.e., hit with the lowest E-value; E ≤-10) most often corresponded to sequences derived from the Insecta with 225 ESTs (69%) showing greatest similarity to *T. castaneum*, followed by 8 ESTs (2%) for *Drosophila* spp., and 6 ESTs (2%) for *Apis mellifera*. Of the remaining ESTs, 63 (19%) showed greatest similarity to coleopteran species other than *T. castaneum*, 12 (4%) to non-coleopteran insect species, and 14 (4%) most closely resembled sequences derived from non-insect source material. Sequences with a significant hit were further characterized using controlled vocabularies using Gene Ontology. Overviews which include hierarchical listings of associated molecular functions, biological processes, and cellular components are provided in Tables 2, 3, and 4, respectively.

### Transcripts putatively associated with *A. glabripennis* development and metamorphosis

Table 5 highlights a subset of developmental and metamorphosis-related transcripts identified in the *A. glabripennis* library. A brief discussion illustrating the role(s) of several of these transcripts is offered below along with select references.

### Autophagk cell death

WHALB004–85 and WHALB007–57 encompassed the complete coding domains of a putative peptidyl-prolyl cis-trans isomerase (PPIase) and eukaryotic translation initiation factor. Using serial analysis of gene expression (SAGE), Gorski et al. (2003) substantiated the involvement of equivalent proteins (e.g., Dmel\cypl and Dmel\eIF-5A) in authophagic cell death. While generally considered as a defense mechanism, this process is believed to be imperative for organelle turnover and recycling during the transition from late instar/pre-pupa to pupa in holometabolous insects such as *A. glabripennis*.

**Table 2.**
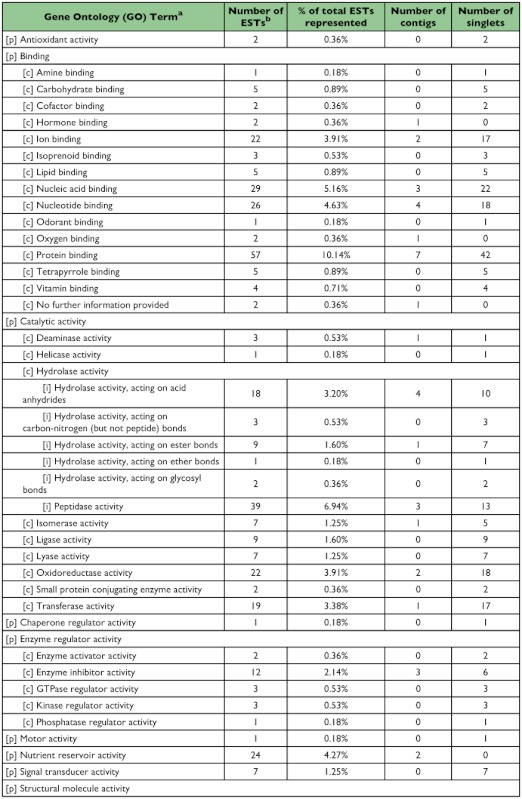
Molecular function

**cont:**
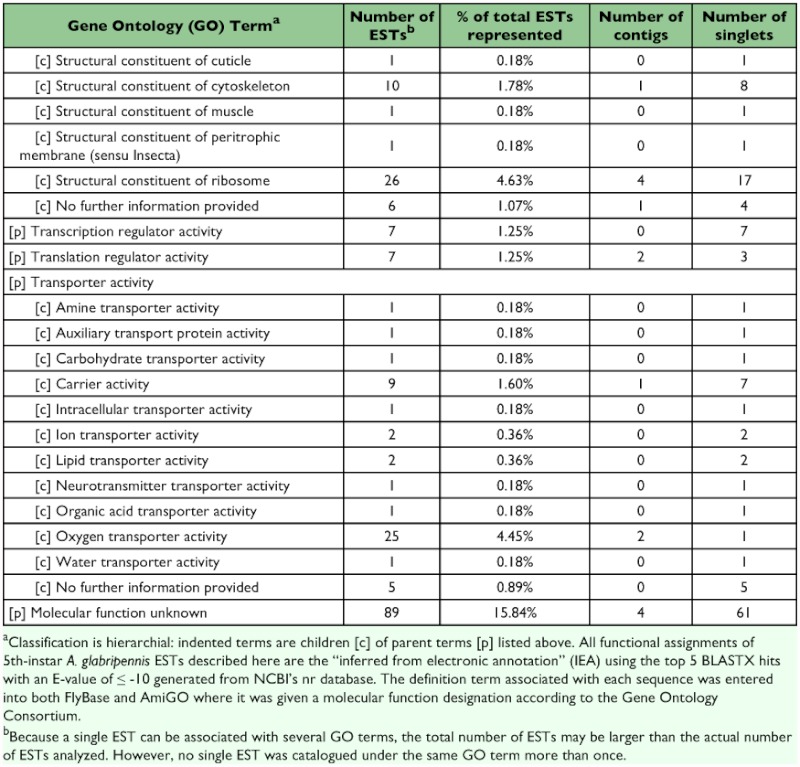

### Bristle morphogenesis

Singletons WHALB002-36 and WHALB004-32 corresponded to *D. melanogaster* singed [CG32858-PA, isoform A] and darkener of apricot (Doa) [CG33553-PE, isoform E]. Although most often associated with neurosensory bristle development, these proteins are thought to be critical in an array of developmental processes including antennal morphogenesis, compound eye development, salivary gland autophagic cell death, and sex differentiation (Yun et al. 1994).

### Nervous system development

Analysis of the primary sequence of WHALB[0248] exposed what appears to be a “false contig” (i.e., product of two distinct transcripts erroneously conjoined through alignment of analogous sequence). The contig was subsequently dissolved and each EST compared to the nr database separately. Based on results of the query, WHALB007-9 was retained along with the BLASTX match definition listed in Table 5. Because WHALB007-9 and WHALB[0269] were assigned equivalent designations, it was necessary to ascertain whether these sequences could be assembled using less stringent parameters. However, superposition of the translated sequences to *D. melanogaster* CG4264-PA, isoform A isoform 1 revealed an 8 amino acid gap corresponding to Dmel TQASIEID_278–285_ that failed to link the *A. glabripennis* sequences. While not contiguous, these assembled sequences represent transcripts that putatively encode heat shock protein cognate 4 (Hsc70-4), a protein which functions in nerve projection events such as axon guidance, axonal fasciculation, neurotransmitter secretion and synaptic vesicle transport.

**Table 3.**
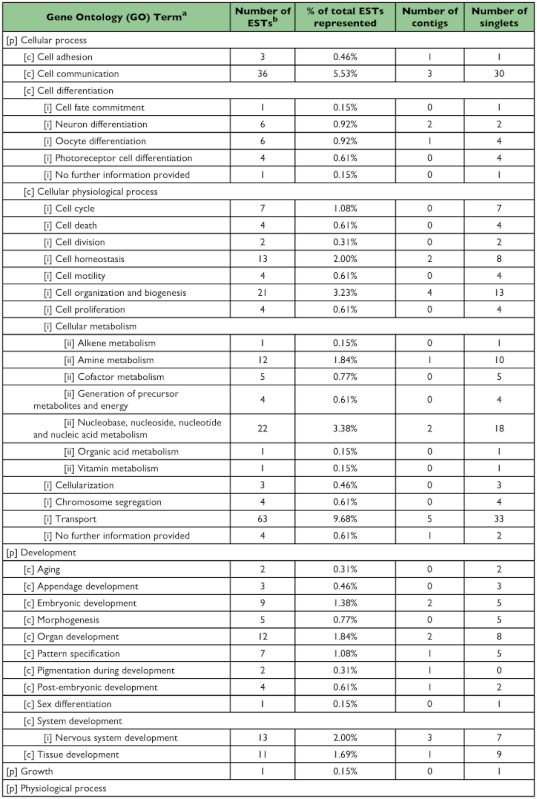
Biological process

**cont:**
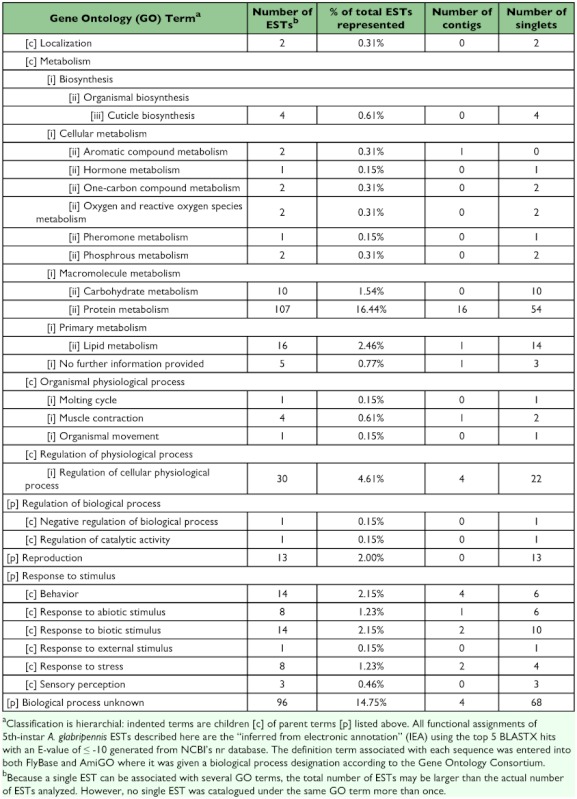

**Table 4.**
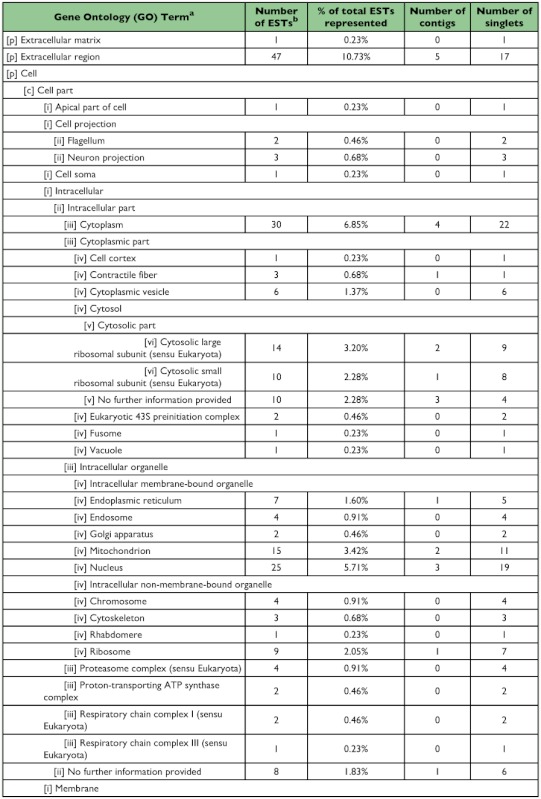
Cellular component

**cont:**
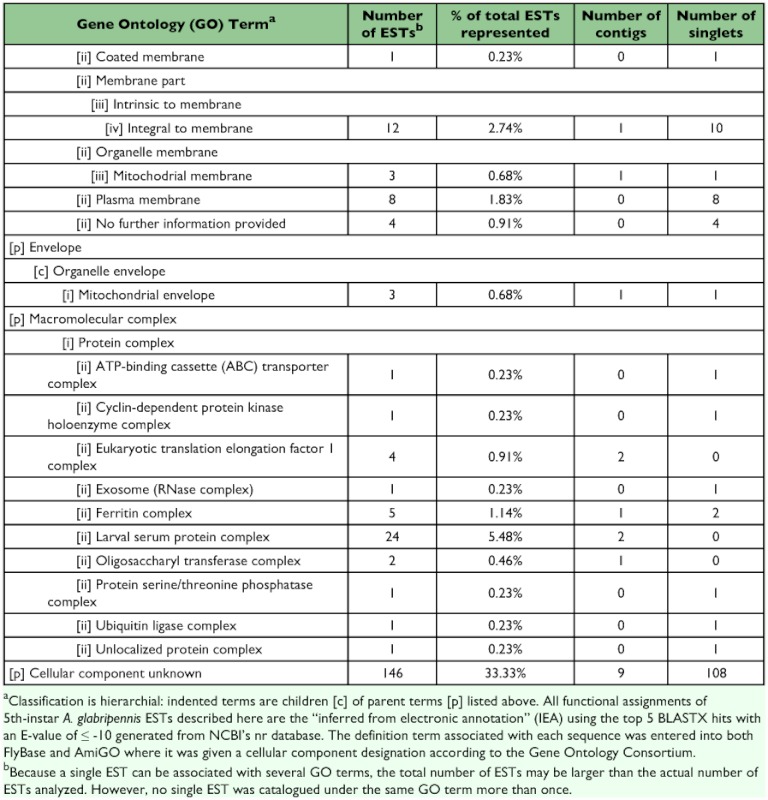

WHALB [0262] consisted of a single ORF containing the entire coding domain of a putative protein paralleling *D. melanogaster* ciboulot (cib). Like Dmel\cib, the translated sequence of the *A. glabripennis* coding domain is highly congruent, at least on an amino acid level, to β-thymosins (e.g., *Bombyx mori* thymosin isoform 2, 5.00E-40; accession no. ABF51487). In particular, an actin binding motif found in both β-thymosins and cib was identified as KLKHTLTQEK_74–83_ within the WHALB [0262] ORF (Nachmias 1993). However, as observed in Dmel\cib, Agla\cib may possess biochemical properties comparable to profilin rather than thymosin with binding to monomeric actin occurring exclusively at the barbed (or plus) end of the filament and enhanced actin-based motility observed *in vitro* (Loisel et al. 1999). This regulation of actin assembly is thought to be a key factor governing axonal outgrowth during the differentiation events that underlie brain metamorphosis (Boquet et al. 2000).

### Muscle development

WHALB[0331] and WHALB003-14 were catalogued under the transcript class “muscle development”. Although annotated based on non-traceable author statements listed in either NCBI's GenBank or FlyBase, these assembled sequences clearly possess sequence similarity to commonly accepted muscle-associated proteins such as muscle protein 20-like protein and muscle LIM protein.

### Cuticle development and puparium formation

Transcripts that potentially code for proteins involved in cuticle biosynthesis were also identified within the *A. glabripennis* library. For example, WHALB002-14 showed significant sequence similarity to the coat protein complex (COPII) small G protein Sar l. In 2005, Abrams and Andrew found that mutations of this gene in *Drosophila* resulted in a range of cuticle defects including reduced cuticle length and pigmentation as well as changes in ventral dentricle and dorsal hair morphology.

**Table 5.**
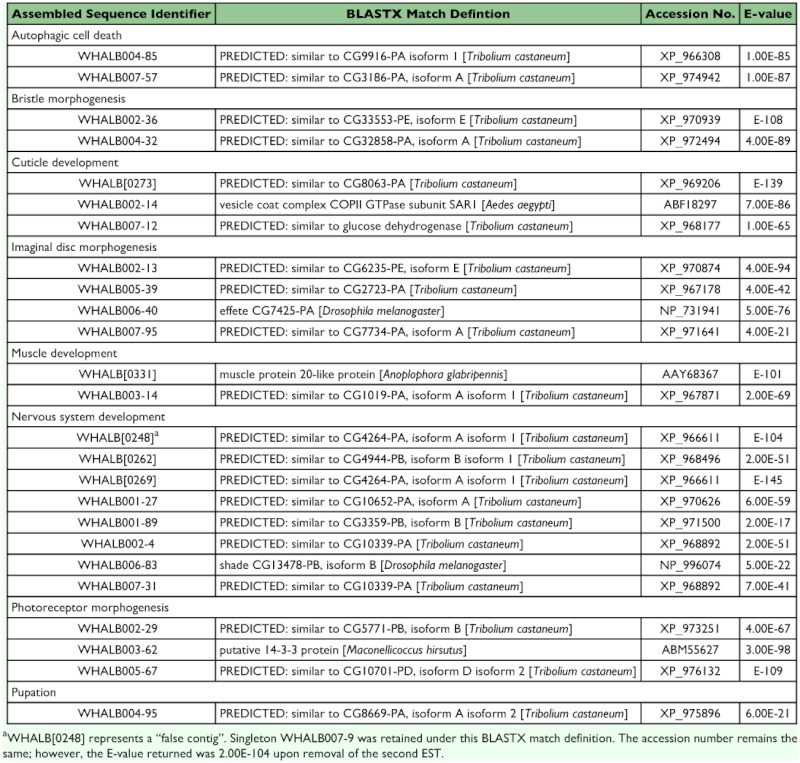
Transcripts putatively associated with A. glabripennis development and metamorphosis

Two ESTs aligned to form WHALB[0273], a contiguous sequence with homology to *D. melanogaster* yellow-f2 [CG8063-PA]. This enzyme plays a major role in melanization reactions that may contribute to sclerotization/ tanning of the late stadia or adult insect cuticle (Han et al. 2002). In addition, WHALB004-95 and WHALB007-12 returned matches to cryptocephal (crc) [GG8669-PA, isoform A isoform 2] and glucose dehydrogenase (Gld), respectively. Gene expression and deletion studies have shown that both Gld and crc act either in response to the late larval ecdysteroid pulse or in the regulation of ecdysone biosynthesis/secretion during the onset of pupariation (Andres et al. 1993; Hewes et al. 2000).

### Imaginai disc morphogenesis

WHALB002-13 closely resembled *D. melanogaster* twins (tws) [GG6235-PA, isoform A] with a 90% identity and 97% positives. This gene product was originally discovered via a P-element mutation that induced the formation of extra anlagen in the posterior compartment of the wing disc of *Drosophila* (Uemura et al. 1993). This phenomenon of precursor duplication illustrates the importance of phosphorylation and dephosphorlyation events in the regulation of tissue pattern specification not only in relation to imaginal disc morphogenesis, but also in regards to several other crucial developmental processes including maturation of the peripheral nervous system and determination of photoreceptor fate in the compound eye. In 2004, Bajpai et al. further demonstrated that Dmel\*tws*^j11C8^, which codes for the B/PR55 regulatory subunit of protein phosphatase 2A (PP2A), functions as a positive regulator of Wg/Wnt signaling. This signal transduction pathway was also linked to singletons WHALB001–37, WHALB004–34, and WHALB00–94, although their role(s) in insect development may involve alternate biological processes such as fatty-acid/retinoid binding and lipid transport.

### Photoreceptor morphogenesis

WHALB002–29 possessed sequence similarity to *D. melanogaster* Rab 11 [GG5771-PB, isoform B], a small GTPase implicated in a variety of trafficking events associated with photoreceptor terminal differentiation including colocalization with rhodopsin at the base of the rhabdomere, formation of multivesicular body (MVB) endosomal compartments, and development of specialized structures within Garland cells (Satoh et al. 2005). Likewise, WHALB005–67 returned a significant BLAST hit to Moesin, an integral component in *Drosophila* photoreceptor morphogenesis. Although the singleton represented only a partial coding domain, query of the translated sequence using RPS-BLST revealed a portion of the N-terminal FERM domain (FERM_C) confirming its placement within the Ezrin-Radixin-Moesin (ERM) family of proteins. While these proteins are broadly associated with actin-based scaffolding, gene disruption studies involving RNAi and loss-of-function mutations in *Drosophila* have suggested that Dmel\VMoe, in particular, is essential for proper assembly of the apical membrane skeleton that supports the microvillar array of the rhabdomere (Karagiosis and Ready 2003).

## Conclusions

This study represents the first investigation regarding the transcriptome of *A. glabripennis*. The resultant sequence data has been made available to the public and has been catalogued according to a controlled vocabulary to facilitate use of the dataset in future studies. Further, several transcripts have been identified that are specific to *A. glabripennis* that may be involved in growth and morphogenesis. Collectively, these sequences provide a strong foundation for functional genomics studies that will enable the development of more biorational control measures to combat this invasive pest.
